# Classification of COVID-19 electrocardiograms by using hexaxial feature mapping and deep learning

**DOI:** 10.1186/s12911-021-01521-x

**Published:** 2021-05-25

**Authors:** Mehmet Akif Ozdemir, Gizem Dilara Ozdemir, Onan Guren

**Affiliations:** 1 Department of Biomedical Engineering, Faculty of Enigneering and Architecture, Izmir Katip Celebi University, 35620 Cigli, Izmir, Turkey; 2 Department of Biomedical Technologies, Graduate School of Natural and Applied Sciences, Izmir Katip Celebi University, 35620 Cigli, Izmir, Turkey

**Keywords:** COVID-19, ECG, Paper-based ECG, GLCM, Hexaxial mapping, Deep learning, Convolutional neural network, Diagnosis

## Abstract

**Background:**

Coronavirus disease 2019 (COVID-19) has become a pandemic since its first appearance in late 2019. Deaths caused by COVID-19 are still increasing day by day and early diagnosis has become crucial. Since current diagnostic methods have many disadvantages, new investigations are needed to improve the performance of diagnosis.

**Methods:**

A novel method is proposed to automatically diagnose COVID-19 by using Electrocardiogram (ECG) data with deep learning for the first time. Moreover, a new and effective method called hexaxial feature mapping is proposed to represent 12-lead ECG to 2D colorful images. Gray-Level Co-Occurrence Matrix (GLCM) method is used to extract features and generate hexaxial mapping images. These generated images are then fed into a new Convolutional Neural Network (CNN) architecture to diagnose COVID-19.

**Results:**

Two different classification scenarios are conducted on a publicly available paper-based ECG image dataset to reveal the diagnostic capability and performance of the proposed approach. In the first scenario, ECG data labeled as COVID-19 and No-Findings (normal) are classified to evaluate COVID-19 classification ability. According to results, the proposed approach provides encouraging COVID-19 detection performance with an accuracy of 96.20% and F1-Score of 96.30%. In the second scenario, ECG data labeled as Negative (normal, abnormal, and myocardial infarction) and Positive (COVID-19) are classified to evaluate COVID-19 diagnostic ability. The experimental results demonstrated that the proposed approach provides satisfactory COVID-19 prediction performance with an accuracy of 93.00% and F1-Score of 93.20%. Furthermore, different experimental studies are conducted to evaluate the robustness of the proposed approach.

**Conclusion:**

Automatic detection of cardiovascular changes caused by COVID-19 can be possible with a deep learning framework through ECG data. This not only proves the presence of cardiovascular changes caused by COVID-19 but also reveals that ECG can potentially be used in the diagnosis of COVID-19. We believe the proposed study may provide a crucial decision-making system for healthcare professionals.

**Source code:**

All source codes are made publicly available at: https://github.com/mkfzdmr/COVID-19-ECG-Classification

## Background

Coronavirus Disease 2019 (COVID-19), caused by the novel coronavirus severe acute respiratory syndrome coronavirus 2 (SARS-CoV-2), first emerged in the Wuhan region of China in early December 2019. COVID-19 is a contagious virus that causes respiratory tract infection and can be transmitted from person to person and it has continued to spread since its first appearance and caused a pandemic that still continues around the world [1, 2]. It has been affecting life negatively in terms of health, economy, and social aspects [3]. As of March 3, 2020, the global mortality rate is 3.4%. As of May 5, 2021, there are more than 153 million confirmed cases. Over 132 million people have recovered, while more than 3.2 million patients died due to the virus [4]. Fast and accurate diagnosis of the disease is of great importance in this process. For this reason, various protocols for the diagnosis of the disease have been announced by the World Health Organization (WHO). Today, the most widely used standard test method for diagnosing COVID-19 is real-time reverse transcriptase-polymerase chain reaction (rRT-PCR). Although PCR tests are the gold standard due to the high accuracy rate (sensitivity), they require long waiting times before results (at least 4 to 6 hours), experienced personnel, and a logistically central location [5]. Other tests and diagnostic methods that can produce faster results are still under investigation. One of the methods used for the diagnosis of COVID-19 is radiography images. Due to the disadvantages of the PCR technique, chest radiography images such as computed tomography (CT) and X-ray are frequently used for the early diagnosis of COVID-19 [6]. These images contain useful information for the diagnostic step. Several studies have found changes in radiographic images that were taken before COVID-19 symptoms began [7].

In the fight against COVID-19, Internet of Things (IoT) applications provide great benefits from diagnosis to treatment of the disease [8, 9]. Recent studies suggest to integrate artificial intelligence (AI) into IoT, Industry 4.0, and Industry 5.0 applications to aid healthcare professionals and patients [10–13]. Diagnosis and treatment with AI are frequently used in the field of medicine and it is a helpful tool for clinicians. Deep learning, one of the sub-branches of AI, creates an end-to-end model without the need for manual feature extraction step compared to traditional machine learning methods and it is popularly used in many areas today. As a result of the rapid spread of the COVID-19 pandemic in the world, there are situations where the number of healthcare professionals is insufficient. Due to all these conditions and other disadvantages, interest in AI-based automatic diagnosis systems is increasing day by day. Deep learning methods have the potential to provide timely assistance to patients with the fast and automatic diagnosis of the disease. These methods do not require expertise and therefore they can help healthcare professionals [14].

Many studies have used radiographic images for the diagnosis of COVID-19. Ozturk et al. [14] used X-ray images as input for the deep learning model to diagnose COVID-19 automatically. In the DarkCovidNet model with 17 convolutional layers, they achieved 98.08% accuracy in binary classification (COVID, No-Findings) and 87.02% accuracy in multiclass classification (COVID, No-Findings, Pneumonia) by using the real-time classifier. Toğaçar et al. [15] proposed a model using X-ray images preprocessed with Fuzzy Color for COVID-19 detection via deep learning. They classified the features extracted with MobileNet2 and SqueezeNet using support vector machines (SVM). They achieved 99.72% overall accuracy as a result of multiple classifications (COVID, No-Finding, Pneumonia). Karaknis et al. [16] proposed architecture to create synthetic images to increase the limited number of X-ray images. In their study, with two deep learning models, they used binary classification for COVID-19 and normal cases, and multi-class classification for COVID-19, normal cases, and pneumonia. In the study, the lightweight deep learning model is presented as an alternative to ResNet8. They obtained 98.7% accuracy, 100% sensitivity, and 98.3% specificity for binary classification, and 98.3% accuracy, 99.3% sensitivity, and 98.1% specificity for multi-class classification. For further studies using X-ray and CT images for automatic detection, the reader is referred to the accompanying paper [17]. However, besides the high success rate in diagnosing COVID-19, radiographic imaging techniques have some disadvantages such as not being portable, high cost, large radiation exposure, requiring technical skill for image analysis, and examination [18]. New techniques are needed as the COVID-19 pandemic continues.

While the primary impact area of COVID-19 infection is the respiratory system, it also affects multiple human body systems, especially the cardiovascular system [19]. The cardiovascular changes in COVID-19 patients [20–24] have prompted an investigation of the diagnostic value of the electrocardiogram (ECG). In the literature, many types of cardiovascular changes in COVID-19 which can be classified as cardiac arrhythmias, QRST abnormalities, myocarditis and pericarditis, and conduction disorders were reported [25]. The most important finding in ECGs of COVID-19 patients is the ST changes [21, 22, 26–31]. Shortening of the PR interval [29, 32] and changes such as QT prolongation [33–37] were also observed in the ECG of COVID-19 patients. It should be noted that some studies claim that COVID-19 cannot be considered the complete cause of these cardiovascular complications, but it should be emphasized that it can reveal the underlying conditions or worsen them [25].

Considering the published studies, ECG can be used to evaluate mortality, intubation, and intensive care unit entry rates beyond patients with severe disease. In order to propose ECG as a diagnostic assessment of COVID-19, ECGs of the moderate and asymptotic patients need to be analyzed. Recently, a research group has published a publicly available dataset containing paper-based ECG of normal (no cardiac findings), cardiac and COVID-19 patients, which provides an opportunity to succeed in the proposed aim. Considering the advantages of ECG application such as low cost, harmlessness, accessibility, and real-time monitoring, automatic detection from ECG may be of great value in COVID-19 diagnosis besides radiography images and PCR. In the previous researches, no studies have been found in which deep learning or even AI is applied using ECG data to the diagnosis of COVID-19, to the best of our knowledge.

Additionally, many deep learning approaches were proposed for automatic cardiac arrhythmia detection. Besides using 1D ECG signals [38, 39] to train the deep network, in many studies were used a 2D representation of 1D ECG signals like ECG time-amplitude images [40–43], time-frequency representations by using Short-Time Fourier Transform (STFT) [44, 45] and Continuous Wavelet Transform (CWT) [46], higher-order spectral representations [47], and dual beat coupling matrices [48] in order to train CNN architecture. Considering the wide usage of paper-based ECG reports [49], there is a lack in the automatic detection of cardiac problems which require special attention.

In the light of these findings, this study addresses two different problems:

- Automatic classification of the disorders that may occur in ECG due to COVID-19 and even automatic diagnosis of COVID-19 through ECG data.
- In cases where ECG data can be collected in the form of paper-based reports instead of digital ECG signals, proposing a novel and effective method that can aid automatic diagnosis from printed paper-based ECG reports.

For these purposes, a novel, low-cost, and efficient automatic COVID-19 diagnosis method is proposed for the first time using deep learning and hexaxial feature mapping with ECG data in this study. Firstly, paper-based ECG images obtained from the publicly available database are pre-processed and segmented. Then a novel hexaxial feature mapping process is implemented to generate hexaxial ECG images. These hexaxial ECG images are trained with a new deep network architecture to diagnose COVID-19.

In the following, this paper is structured as; in the Related works section, related papers investigating cardiac findings that may be caused by COVID-19 are summarized; in the Methods section, firstly, the used dataset is explained, then the segmentation and pre-processing of the paper-based ECG images, feature extraction step, ECG mapping process, and finally the proposed deep network architecture are examined in detail; in the Results and discussion section, the classification results are presented, and findings and limitations are discussed; and finally in the Conclusion section, the main findings of the study are summarized and some useful suggestions are given.

## Related works

In this section, the changes seen in ECG associated with COVID-19 are detailed with the studies in this field. Wang et al. [33] detected abnormal ECG in 201 of 319 COVID-19 patients and they reveal that ST-T change is the most important clinical evidence in the abnormal ECG. In addition, sinus tachycardia, atrial arrhythmia, right bundle branch block (RBBB), sinus bradycardia, atrial fibrillation (AF), atrial tachycardia, abnormal Q-wave, and weak R-wave progression were also observed in the ECG of patients with COVID-19. In the comparative statistical analysis between patients with and without the severe disease, a significant difference was found in all complications. A significance of $p < 0.001$ was achieved in the ST-T change. Pavri et al. [32] tried to detect heartbeat and PR changes from the ECG of 75 COVID-19 patients. In 50.7% of patients with COVID-19, it was observed that the PR interval shortened with the acceleration of the heart rate. In addition, no change was observed in 49.3% of COVID-19 patients. In the statistical analysis performed with ECGs taken before COVID-19 and during COVID-19, a significant difference was found between the two groups in their heart rate and PR interval. In the conducted study, the mortality rate was found to be higher in patients with shortened PR interval. Angeli et al. [50] examined the ECGs of 50 patients with COVID-19. They found ST-T abnormality in 30% of the patients and left ventricular hypertrophy in 30%. Also, various abnormalities such as AF, tachy-brady syndrome, and acute pericarditis have been detected in the ECG of patients with COVID-19 during hospitalization. Although rare, RBBB and Myocardial Infarction (MI) have been observed in patients with COVID-19. Li et al. [51] conducted a study by examining the ECG of 113 COVID-19 patients 50 of whom died and 63 of whom survived. Ventricular arrhythmia was found to be statistically significant evidence in patients who died compared to patients who survived. In addition, sinus tachycardia was observed widely in the ECG of patients with COVID-19. Santoro et al. [34] detected QT prolongation in 14% of the patients in their study, by examining the ECG of 110 patients with COVID-19. Jain et al. [35] reported that the drugs used for the treatment of COVID-19 caused QT prolongation in the ECG. To test this situation, the ECG of 2006 COVID-19 patients was examined. According to the obtained results, QT prolongation was detected in 19.7% of patients with COVID-19. In addition, it was determined that patients with this abnormality in their ECGs had higher rates of intubation and intensive care unit entry than others. Mccullough et al. [52] evaluated whether the ECG provides prognostic information in COVID-19 disease. In their study, they examined the ECG of 756 patients with COVID-19 and detected abnormalities such as atrial premature contractions, intraventricular block, repolarization abnormalities, and RBBB. Among these findings, ST-elevation was rarely observed. And it was stated that patients with these ECG findings had higher mortality rates. Lam et al. [29] conducted a study with 18 COVID-19 patients. They detected abnormalities including PR depression, biphasic T-waves, PR prolongation, Q-waves, ST-segment elevation, atrial flutter, RBBB, and atrial trigeminy in 63% of the patients. According to their results, it was determined that COVID-19 patients with abnormal ECG tended to have increased severity and stay in the hospital for 61% longer than other patients. Bertini et al. [30] examined the ECG of 431 patients with COVID-19. They found abnormalities in the ECG of 93% of the patients. AF was observed in the ECG of 22% of patients. Acute right ventricular pressure overload (RVPO) was detected in 30%, and ST-T prolongation was observed in 4 patients. Nemati et al. [53] suggested that ECG could be an early indicator for COVID-19 infection this is because the changes in the ECG were also observed in COVID-19 patients without any cardiovascular history. As detailed above, many studies have demonstrated that some cardiac disorders may be caused by COVID-19 and they can be easily observed in ECG. Also, many cardiovascular changes continue to be associated with COVID-19 infection day by day. Therefore, ECG can be an important diagnostic tool not only for the early diagnosis of COVID-19 but also for the cardiovascular complications which may arise during or after COVID-19 disease for mild patients.

## Methods

**Fig. 1:**
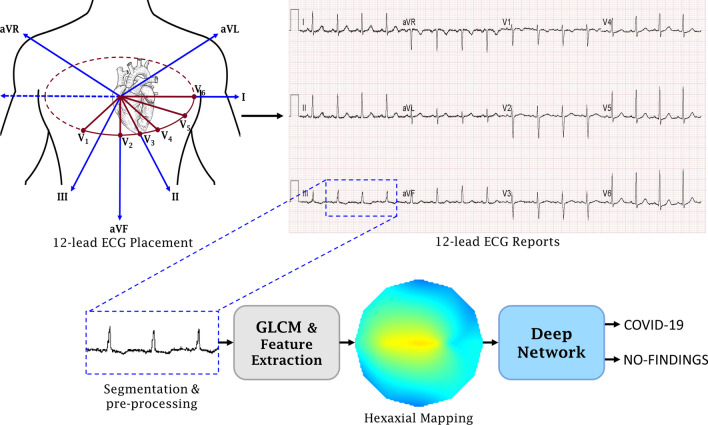
Representation of the proposed framework, includes five-step: **(i)** ECG image dataset acquisition (The heart drawing is provided from a publicly available webpage (Zagrobelna, M.: How to Draw a Heart. Available from: https://design.tutsplus.com/tutorials/how-to-draw-a-heart-cms-30737. Accessed: 2021-05-05).), **(ii)** segmentation, pre-image-processing, and image enhancement, **(iii)** application of GLCM and extractions of some of their properties (includes statistical analysis), **(iv)** ECG hexaxial feature mapping process, and **(v)** designing, training, validating, and testing the proposed deep network (**GLCM: Gray Level Co-occurrence Matrix, paper-based ECG report number: 211 with the label: COVID-19 [54])

This study consists of 5 main stages. The visualizations of these stages are shown in Fig. 1.

### COVID-19 ECG images dataset

In this study, a publicly available dataset containing ECG images of cardiac and COVID-19 patients has been used. The dataset was shared online by Khan et al. [54] from the University of Management and Technology on Mendeley Data. The dataset includes 1937 distinct patients’ paper-based ECG report images. ECG reports were examined by experts and the images consist of 250 COVID-19 patients, 77 MI patients, 548 patients with abnormal heartbeats (recovered from COVID-19 or MI), 203 patients that have MI history, and 859 people without any cardiac findings. The presented dataset is the first dataset shared for the ECG of COVID-19 disease, in fact, it is the first COVID-19 bio-signal database as far as we know.

The paper-based ECG records in the dataset consist of ECG signal drawings from a 12-lead system (I, II, III, aVR, aVL, aVF, V1, V2, V3, V4, V5, and V6) and the sampling rate was 500 Hz. As understood from the paper-based ECG reports, ECG signals were collected via EDAN SE-3 series 3-channel electrocardiograph, and some of the signals were applied with a 0.67–25 Hz bandpass filter, and some of them with a 0.5–100 Hz bandpass filter and a 50 Hz notch filter.

Unfortunately, the images of the dataset have some limitations. The images do not have sufficient resolution, and report image sizes are not standard. In particular, the ECG reports of COVID-19 patients consist of different types of reports. Other reports are more standardized and have better resolution.

In this study, two different classification problems are discussed; (i) *to distinguish* COVID-19 from No-Findings (that have normal ECG); all 250 COVID-19 and 250 out of 859 normal paper-based ECG report images were used and (ii) *to diagnose* COVID-19 (COVID-19 (Positive) versus other types of ECGs (Negative)); all 250 COVID-19, 83 of 859 normal, 83 of 548 abnormal heartbeat and 84 of 280 MI paper-based ECG report images were used. The reason for choosing the equal amount of data in the classification process is to eliminate the imbalanced dataset effect. In addition, all paper-based ECG report images used in this study were selected from the group in which the 0.67–25 Hz bandpass filter was applied. An example for a 12-lead paper-based ECG report of a COVID-19 patient from the dataset (Report number: 211) is shown in Fig. 1.

### Pre-processing and segmentation

In this section, the conversion of noised 12-lead paper-based ECG images to noiseless channel-based binary images is explained. There are many studies that digitize paper-based ECG images [55, 56]. Nevertheless, these studies have high computational costs. Moreover, the complex image processing and digitization processes may cause degeneration of ECG signals and cause information loss. Therefore, in this study, a simple and effective paper-based ECG segmentation approach that does not require any complex image processing method is proposed. Moreover, the proposed method does not involve a digitization process and preserves the ECG signals as images. Hence, no degradation occurs in ECG signals. In the proposed method, the quality of ECG images depends only on the sampling rate of paper-based ECG signals.

**Fig. 2:**
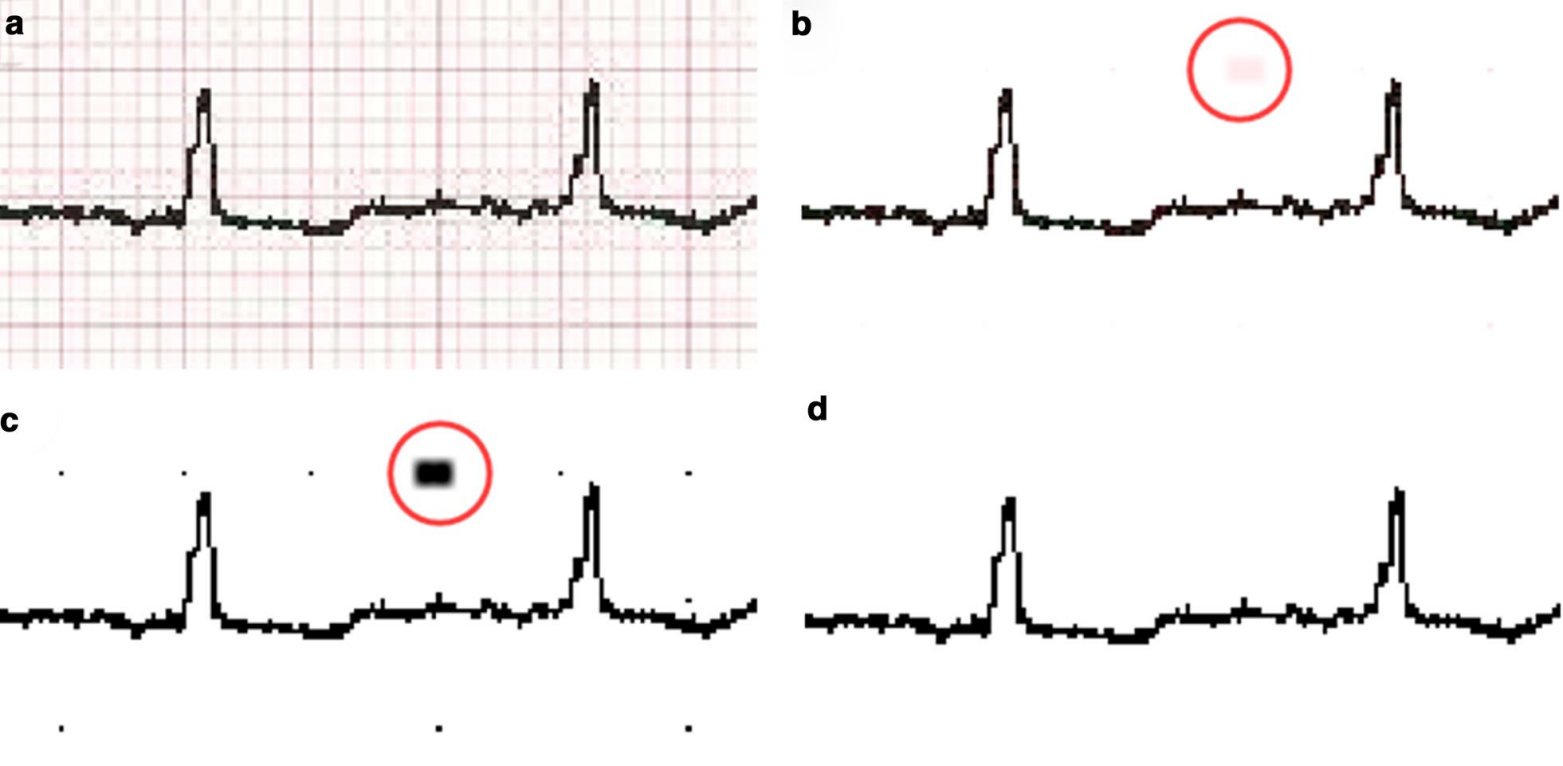
Examples of background removal processes: **a** segmented paper-based ECG image, **b** paper-based ECG image without background lines, **c** binarized paper-based ECG image, and **d** eventual paper-based ECG image

For this purpose, firstly, the part containing each ECG channel is segmented from paper-based ECG images. The segmentation process was carried out with a rectangular frame. Since the paper-based ECG images in the dataset have different resolutions, the positions of this frame on the paper-based ECG image were measured manually. The segmentation process is performed to include one or more RR intervals in each channel. An example of a segmented paper-based ECG image is shown in Fig. 2a. Segmented ECG-channel images have a background sourced from the ECG-paper lines. This background is removed within two steps. In the first step, the background lines were removed by filtering the input densities with a density mapping function [57], because the background has denser or softer RGB values than the curves expressing the ECG signal. This is essentially a contrast enhancement process. An example of a segmented paper-based ECG image with no background lines is shown in Fig. 2b. Unfortunately, the obtained images still include traces of the background where the background lines are as dense as ECG curves. Besides, since only the signal pattern in ECG reflects the information about the heartbeat period, the RGB color distribution of ECG curves is negligible [41, 42]. For this reason, the RGB images obtained in the last stage were converted to binary images by taking the “G” channel as a reference (since “G” channel information is not dominant in paper-based ECG images due to the nature of ECG paper). An example of the paper-based ECG image obtained after the binarization process is shown in Fig. 2c. While the ECG curve consists of adjacent interconnected pixels, background noise is separated from this curve as seen in Fig. 2c. In the second step, the interconnected ECG curve pixels are filtered from background noises by using the *bwareafilt* function of MATLAB^®^. Thus, the eventual binary segmented paper-based ECG image was obtained without any loss. An example of the final image is shown in Fig. 2d. The pre-processed and segmented paper-based ECG image database is available at GitHub.

### GLCM and feature extraction

Feature extraction and selection play an important role in machine learning-based classification problems. A set of images can be categorized according to their most distinctive features which can be found by implementing an appropriate feature extraction method. In our approach, at the end of the pre-processing steps, all paper-based ECG images were converted to binary images where the ECG signal is represented by 0s. We chose the Gray Level Co-Occurrence Matrix (GLCM) method [58] for feature extraction because it has been shown that GLCM is very useful in extracting the important properties of an ECG signal such as periodicity and distortions [59, 60].

GLCM generates a square matrix whose dimension equals the number of gray levels in the image. Each cell of GLCM corresponds to the number of the co-occurring related gray levels in the image. The GLCM matrix *G* can be calculated from the Eq. (1) as given in [60]:

1 $$\begin{aligned} \begin{aligned} G_{\Delta x, \Delta y}(i, j)=\sum _{x=1}^{N} \sum _{y=1}^{M}\left\{ \begin{array}{ll} 1, &{} \quad I(x, y)=i \quad \& \\ &{}\quad I(x+\Delta x, y+\Delta y)=j \\ 0, &{}\quad \text{ otherwise } \end{array}\right. \end{aligned} \end{aligned}$$

where *I* is the image of the pre-processed binary ECG images with dimension *NxM*; *i* and *j* are the pixel values, *x* and *y* are the spatial positions in the image *I*. $\Delta x$ and $\Delta y$ are the spatial offset, and *I*(*x*, *y*) is the pixel value. In our problem, the pixel values *i*, *j* take 0, 1 and *G* is a size of $2\times 2$ matrix. Taking the offset $\Delta x$ and $\Delta y$ values as 1 and 0, respectively, the transitions between the pixel with 0 and 1 intensities in horizontal direction provide the amount of deterioration in the signal specially in its periodicity. The second-order statistical analysis of the GLCM matrix provides different parameters that are widely evaluated as image features in image classification studies [61].

In this work, we extracted the most commonly used four GLCM features which are energy, contrast, correlation, and homogeneity from each lead of the segmented binary ECG images. Mentioned features can be calculated using the *G* matrix obtaining in Eq. (1) as follows:

2 $$\begin{aligned} \begin{aligned} Energy&= \sum _{i=0}^{1} \sum _{j=0}^{1} p_{ij}^2\\ Contrast&= \sum _{i=0}^{1} \sum _{j=0}^{1}(i-j)^2 p_{ij}\\ Homogeneity&= \sum _{i=0}^{1} \sum _{j=0}^{1} \frac{1}{1+(i-j)^2}p_{ij} \\ Correlation&= \sum _{i=0}^{1} \sum _{j=0}^{1} \frac{(i-\mu _i)(j-\mu _j)}{ \sigma _i \sigma _j} p_{ij} \end{aligned} \end{aligned}$$

where $p_{ij}$ is the probability of adjacent pixels that have *ij* intensity pattern and it is stored in the GLCM matrix *G*, i.e. for a binary image first element of *G* shows the probability of co-occurrence 00 pattern in the image *I*. $\mu _i$, $\mu _j$, $\sigma _i$, and $\sigma _j$ are means and standard deviations of the intensities, and were given as follows:

3 $$\begin{aligned} \begin{aligned} \mu _i&= \sum _{i=0}^{1} \sum _{j=0}^{1} i p_{ij}&\sigma _i&= \sqrt{\sum _{i=0}^{1} \sum _{j=0}^{1} (i-\mu _i)^2 p_{ij}} \\ \mu _j&= \sum _{i=0}^{1} \sum _{j=0}^{1} j p_{ij}&\sigma _j&= \sqrt{\sum _{i=0}^{1} \sum _{j=0}^{1} (j-\mu _j)^2 p_{ij}} \end{aligned} \end{aligned}$$

**Fig. 3:**
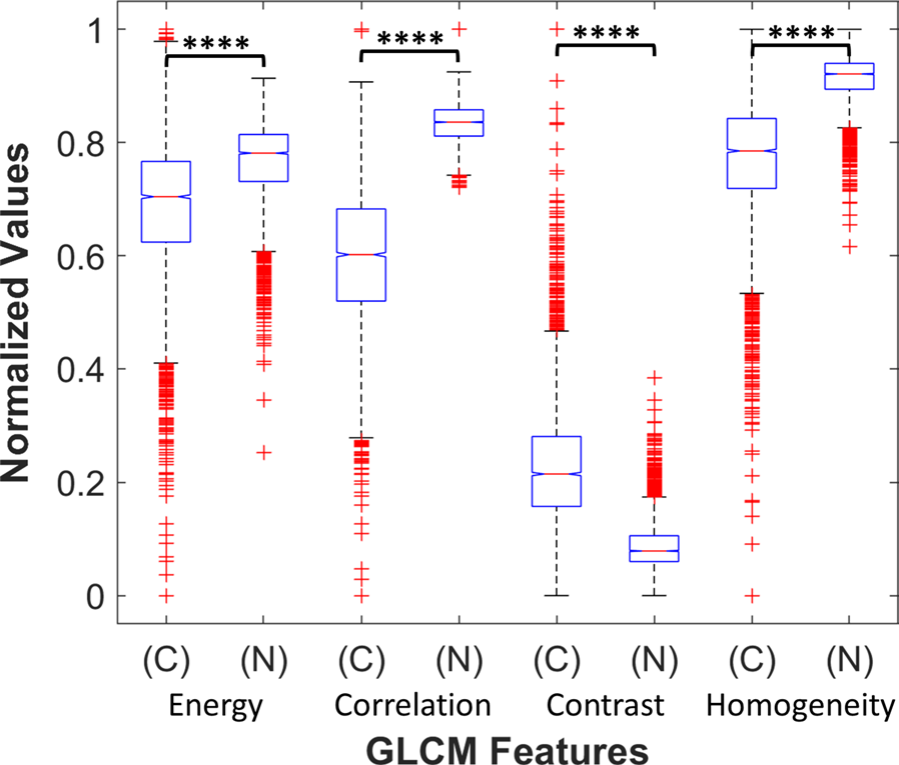
One-way ANOVA results within a box plot for each related GLCM features. All normalized GLCM features obtained from binary ECG images were verified to be statistically significant relative to each other ($p < 0.0001$ for all binary groups). Each group has a total of *4500 samples: 18-lead* *x* *250 paper-based ECG reports* (C: COVID-19, N: No-Findings)

We assessed the four GLCM features from a statistical perspective in order to select the most informative and distinctive feature to represent the binary ECG images. We performed the one-way ANOVA test on GLCM features obtained from the binary ECG images. ANOVA test results of GLCM features that belong to No-Findings and COVID-19 classes are given in Fig. 3. We found that all GLCM features were statistically significant relative to each other ($p < 0.0001$). When the gray level pixel distribution of an image is periodic or homogeneous, the energy value is expected to converge to the upper limit [62]. We concluded that it is prominent to use GLCM energy among the other GLCM features to emphasize the periodicity relation between RR intervals in ECG images. In addition, the energy values are directly related to uniformity. As explained in the Related works section, since the periodicity and orderliness of COVID-19 ECG images are expected to be different from the ECG images without COVID-19, it has become important to measure image uniformity. Moreover, GLCM energy values help determine disorders in texture [63] which may be related to COVID-19. Although all GLCM features that are obtained are statistically significant, for the reasons explained above, the GLCM energy is chosen as a feature to be used in the mapping process.

### Hexaxial feature mapping

Inspired by our previous study [64], we proposed a novel method to represent the paper-based ECG record as a colorful two-dimensional image for various deep learning applications. The feature mapping approach can be defined as assigning a specific value to a specific point in a two-dimensional space. The point here is the projection of the measurement location in three-dimensional space into two-dimensional space (which we call the image plane in our study). The value is the feature that represents the measured signal in the related measurement point, i.e. the GLCM energy of binary ECG images of each lead. We used the hexaxial diagram of heart’s electrical axis [65] as the image plane to define the measurement points.

**Fig. 4:**
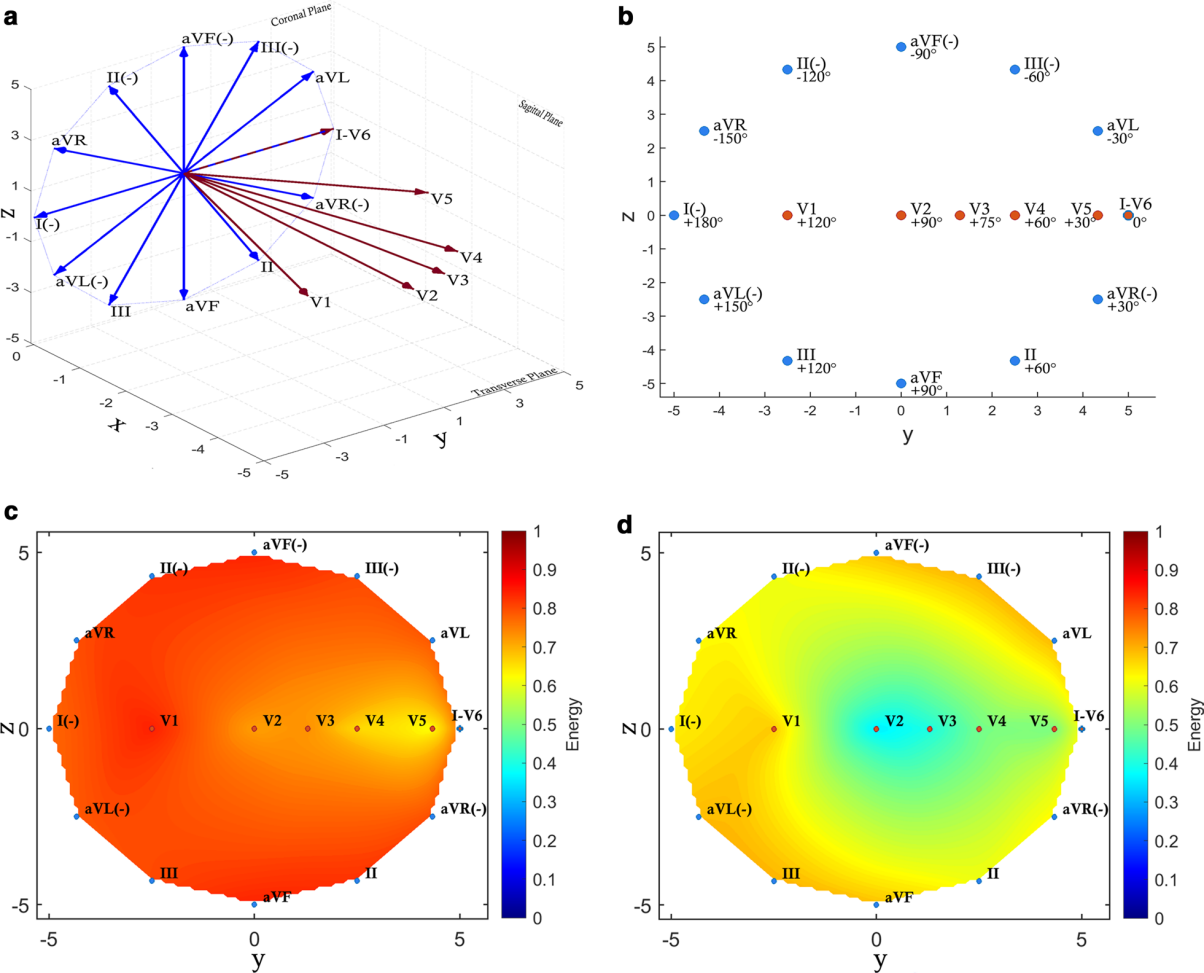
ECG electrode location representations: **a** 3D representation of hexaxial and horizontal reference systems of 12-lead ECG acquisition, **b** 2D mapping of 3D hexaxial (limb leads) and horizontal (precordial leads) reference systems on the coronal plane; and an example of hexaxial feature mapping by using GLCM energies for **c** No-Finding class (Report Number: 182) and **d** COVID-19 class (Report Number: 16)

Our method relies on the 12-lead ECG record system which is accepted as the gold standard for ECG diagnosis, and works with the logic of combining Einthoven, Goldberger, and Wilson derivation systems [66]. In Fig. 4a, 6 limb leads (blue arrows) (I, II, III, aVR, aVL, aVF), their reversed polarities (− I, − II, − III, − aVR, − aVL, − aVF), and 6 precordial leads (red arrows) (V1, V2, V3, V4, V5, V6) are shown. In ECG analysis, the projection of six limb leads with their negative poles on the coronal plane is called a hexaxial reference system shown by the blue points in Fig. 4b. In this presentation, lead I is selected as the zero reference point and lead I and aVF intersect at a right angle at the electric center of the heart. The precordial leads have lied on the transverse plane and only the positive pole of each lead is indicated by the end labels shown by the red points in Fig. 4b. It is assumed that the Lead V6 is parallel to Lead I and the other precordial leads must be placed with a phase angle from V6 in the transverse plane. The leads V2 and V6 intersect at approximately a right angle at the electrical center of the heart [67]. The phase angles of all leads are given in Fig. 4b.

According to this configuration, the heart is assumed to be placed at the origin of a 3D cartesian coordinate (*x*, *y*, *z*) system with axis Lead I (or V6) as *y*, aVF as $-z$ and V2 as *x*. Lead I and aVF span the coronal plane whereas V6 and V2 span the transverse plane. To find measurement points of all leads in 3D space, we assume that the endpoints of each limb leads lie on a circle centered at the origin with a radius *r* in the image plane, and the endpoint of precordial leads lie on a semi-circle centered at the origin with a radius *r* in the transverse plane. The measurement points of the **limb leads** are already in the image plane and they can be calculated easily using these transforms:

4 $$\begin{aligned} \begin{aligned} x&= 0 \\ y&= r\cos {\theta } \\ z&= r\sin {\theta } \end{aligned} \end{aligned}$$

where $\theta$ denotes the given phase angles of the leads. The measurement points of the **precordial leads** lie on the $x-y$ plane and can be projected onto the image plane by using these transforms:

5 $$\begin{aligned} \begin{aligned} x&= 0\\ y&= r\cos {\theta } \\ z&= 0 \end{aligned} \end{aligned}$$

As a result, 18 measurement points are defined in 2D cartesian coordinate $(y-z)$ system. The virtual measurement points and their placement in the image plane are shown in Fig. 4b.

The next step in the hexaxial feature mapping method is assigning a value to the measurement point that represents the measured signal. The hexaxial feature mapping procedure can be defined as follows:

6 $$\begin{aligned} HFM(y_{{\rm lead}},z_{{\rm lead}})=\left\{ \begin{array}{ll} E(I_{{\rm lead}}), & \text{lead} = \text{I, II, III,}\\ & {}\text{aVR, aVL, aVF,} \\ & {} \text{V1, V2, V3, V4, V5, V6} \\ E({\overline{I}}_{{\rm lead}}), &\text{lead} = -{\text{I}}, -{\text{II}}, -{\text{III}},\\ \quad &-{\text{aVR}}, -{\text{aVL}}, -{\text{aVF}} \end{array}\right. \end{aligned}$$

where *y*, *z* shows the location of the projected measurement points of the leads, and *E* indicates the normalized GLCM energy (Energy values rescaled between 0 and 1 to avoid inconsistency and bias.). *HFM* is an expression of the value of *E* at location *y*, *z*. The *HFM* of the positive poles of limb leads and the precordial leads are found by calculating the GLCM energy of binary ECG images denoted by *I*. To find the *HFM* of the negative poles, the images of corresponding positive poles are vertically mirrored denoted by ${\overline{I}}$ then the GLCM energy is computed.

As a result, the GLCM energy features of each lead are mapped onto the coronal plane using virtual measurement points in the 2D plane. A natural two-dimensional neighbor interpolation process [68] is carried out between the existing measurement points to generate a smooth 2D colorful image. In Fig. 4c (no cardiac findings) and Fig. 4d (COVID-19) RGB color representation of the hexaxial feature mapping images are shown. When these two images are compared, it can be seen that the hexaxial feature mapping method has succeeded in representing the ECG of a healthy person in a distinguishing way from the ECG of COVID-19 patients. Furthermore, the proposed approach not only provides a 2D image representation for deep learning studies but also collects all 12-lead ECG information into a single image. Thus, the information contained in the multi-channel ECG can be analyzed and processed over a single image. Since the proposed approach includes the derivation information of 12-lead ECG, hexaxial mapping images also contain the electrical axis activity of the heart.

### Proposed deep learning architecture

Recently, Convolutional Neural Network (CNN) architectures have become incredibly popular in image classification, object detection, and segmentation. A typical CNN architecture consists of a convolutional layer, a pooling layer, and a fully connected layer, respectively. The CNN architecture aims to obtain deep features. The convolutional layer scans the inputs via a filter and obtains feature maps. The pooling layer provides the selection of more meaningful features to reduce the computational cost. And finally, the fully connected layer flattens the inputs and calculates the probabilities of the labels. There are many CNN architectures proposed for image classification consisting of the combination of these layers. Designing a model inspired by previously proven CNN models is more efficient than rebuilding a new architecture [14].

**Fig. 5:**
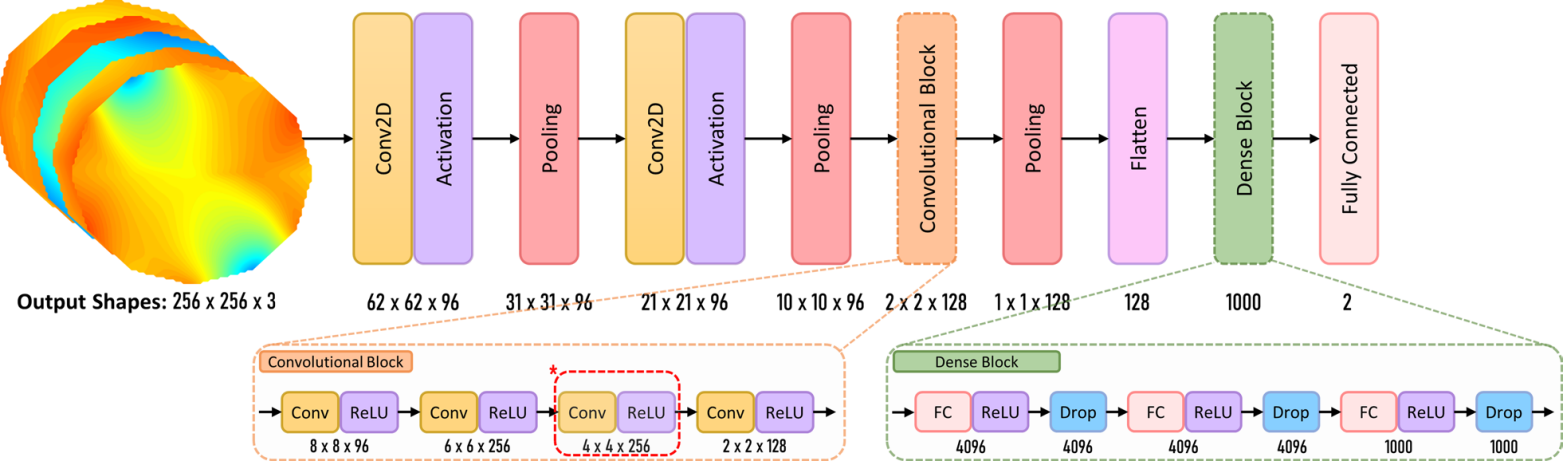
Graphical representation of proposed architecture (Conv: Convolution, FC: Fully Connected, ReLU:Rectified Linear Unit)

In recent studies, various approaches were conducted on the selection of deep network architecture [14, 16, 69, 70]. Ardakani et al. [71] trained 10-different CNN architectures and emphasized the network which had the best classification performance among trained models. In [69], ResNet-50 is selected as a base model due to it yielded the best classification performance. In this study, two main criteria were considered to build the deep model; computational complexity and classification performance. For this purpose, hexaxial feature mapping images were trained with the network architectures which are suggested by recent state-of-the-art studies. When the classification results are compared ResNet-50 [72], AlexNet [73], ResNet-8 [16], and SqueezeNet [74] yielded an accuracy of 73.65%, 93.60%, 85.12%, and 92.52%, respectively. The results revealed that AlexNet which has less network depth achieved significant accuracy compared to well-known architectures. Additionally, the AlexNet model was presented, it was used to classify about 1.2 million images in 1000 different classes. Besides, AlexNet used the Dropout method to overcome overfitting and Rectified Linear Units (ReLU) as the activation function to shorten the training time. Also, the model was compatible with multiple GPUs. Due to these advantages, AlexNet achieved the best performance in ImageNet Large Scale Visual Recognition Challenge in 2012 (ILSVRC2012) [73]. Further, AlexNet has achieved effective performance in many ECG classification studies [47, 75]. Therefore, a new deep network architecture modified from the AlexNet model is designed in this work. Graphical representation of the proposed CNN architecture is shown in Fig. 5.

In the designing phase, we build a 9-layer model that contains one more convolutional layer with 256 filters and ($3\times 3$) kernel size compared to the original AlexNet. Therefore, the input shapes require a larger size of $256\times 256$ compared to AlexNet. Moreover, input images with larger size need to resize, and the padding process is performed on input images that have lower size. In order to avoid increasing the training cost, the number of layers is not increased further. Initially, the high-resolution 300 DPI colorful hexaxial feature mapping images are resized to $256\times 256\times 3$ to feed the proposed CNN architecture. This resizing process also provides less training cost and a balanced kernel size. In the first two layers, the input images are passed through a convolutional layer and a pooling layer. While both layers perform a convolution (Conv) with a ($11\times 11$) kernel and using a ReLU function as the activation function in the convolutional layer, the first one has a stride of 4 and the second one has a stride of 2. Both pooling layers (maximum) used in this step have a kernel size of ($2\times 2$) and a stride of 2. The next stage consists of four repetitive convolutional layers called as a convolutional block. Each convolutional layer has ($3\times 3$) kernels and a stride of 1, and the numbers of filters are 96, 256, 256, and 128, respectively. Following the convolutional block is a maximum pooling layer with ($2\times 2$) kernel size and a stride of 2. In the next step, after the model is flattened, there is a dense block consisting of three fully connected layers. The dropout method (drop rate of 0.4) is used to prevent overfitting after each fully connected layer in the dense block. And finally, the SoftMax function is used as the binary classifier in the output layer. The proposed CNN architecture has over 23.5 million trainable parameters. Also, in Fig. 5 the output dimensions of the network layers are illustrated.

During the training phase of the proposed architecture, Adam Optimizer [76] was used, because of its effective choice of hyperparameters [77]. Moreover, the batch size is fine-tuned with parameter tuning. Different batch sizes (32, 64, 128, and 256) have been tested in the training phase to achieve the least error rate, and the batch size optimized to 128. Furthermore, different learning rates (0.01, 0.001, 0.0001, and 0.00001) were tested to ensure a lower error rate and to prevent saturation of the model. Although decreasing the learning rate hyperparameter slightly increased the training cost, it fine-tuned on 0.0001 to avoid local minimums. Epochs are tuned at 200 to observe the robustness of the models and to compare the test results with equal conditions.

## Results and discussion

In this study, the generated images based on hexaxial feature mapping, explained in the Methods section, are used to train our proposed architecture. All training, validating, and testing phases are performed on a computer with Nvidia GeForce RTX 2080 TI GPU and 64 GB RAM using Tensor Flow 2.2 and Cuda 10.1.

**Fig. 6:**
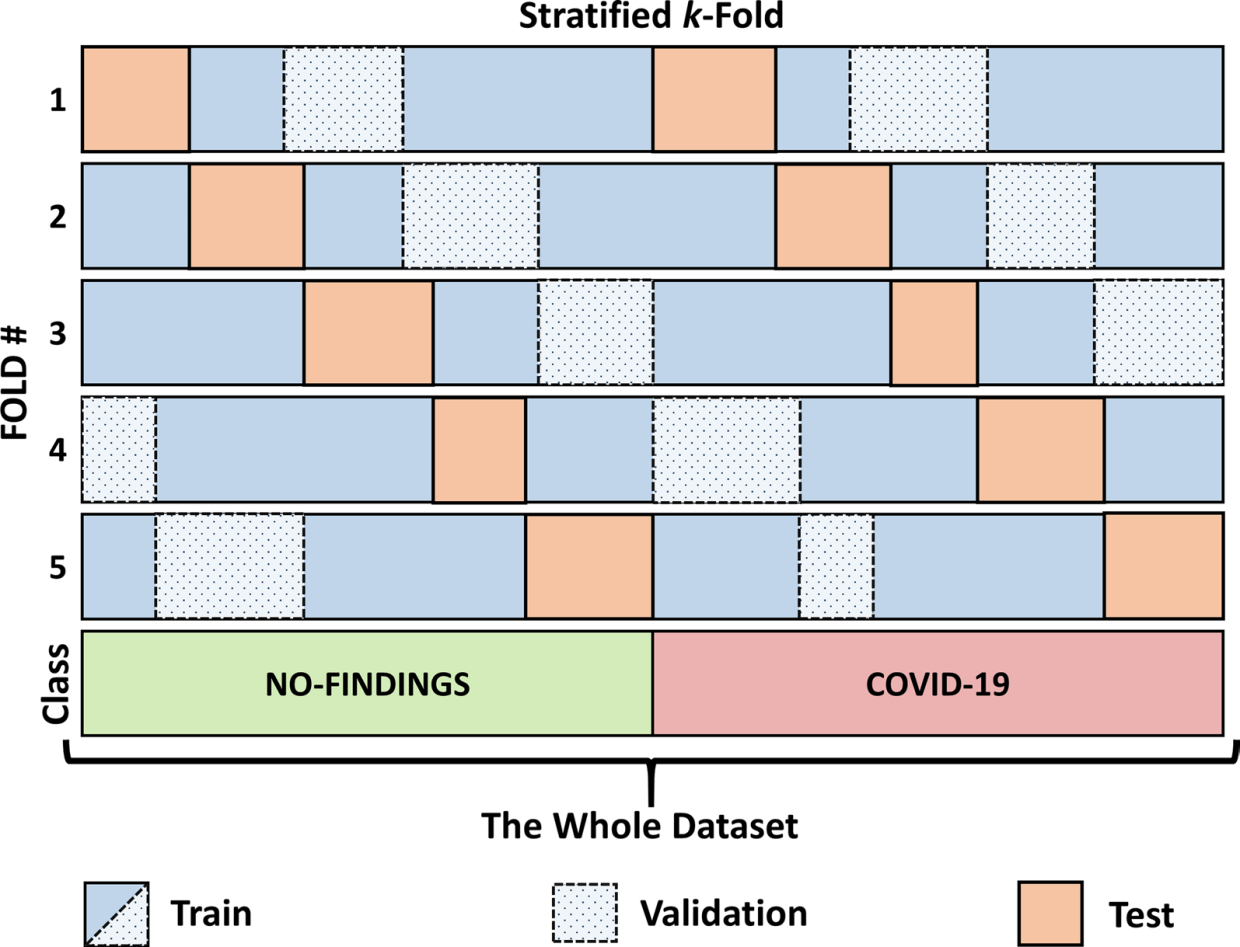
Graphical representation of modified stratified k-fold cross-validation. The number of folds (k) was chosen as 5 in this study. In each fold, the training size, validation size, and test size are 400, 100, and 100, respectively

Modified stratified k-fold cross-validation is adopted to evaluate the robustness of the proposed models in this study. Cross-validation methods are used to evaluate the robustness of models during the training phase. The stratified k-fold cross-validation process re-arranges the dataset to ensure each fold properly represents the entire dataset. We added an extra validation step to the stratified k-fold cross-validation to construct modified stratified k-fold cross-validation which is visualized in Fig. 6. The training phase is processed as follows; firstly, the dataset is shuffled and then split k-part by using the stratified k-fold. While the k-1 part is used to train the model, the remaining one k-part is used in the testing phase and cover all classes without overlap. After the test and training data sets are separated, the remaining training set is split again with a training and validation split process (split rate of 0.25). We chose k as 5 in our study. Considering there are a total of 500 hexaxial mapping images in each phase, 100 images are used in each test phase and any of them are not included in training phase (whereas, the total number of data is 9000 when training 2D ECG spectral images (*18-lead* $\times$ *250 paper-based ECG reports* $\times$ *2 groups*)). The data used in the test phase cover two classes (COVID-19 vs others) with approximately equal amounts of data. The validation data (consisting of 100 images) is used both in the training and validating phases. Thus, a two-step verification process is performed to evaluate the robustness of the models during the training and testing phases. Furthermore, recall (REC), precision (PRE), accuracy (ACC), specificity (SPE), F1-Score (F1-S) [15], area under the receiver operating characteristic curve (ROC-AUC) [64, 78], and mean squared error (MSE) [17] are calculated during the validating and testing phases to investigate the robustness of the models.

### Experimental results and implications

We performed four experiments on two different binary classification problems. All experiments were carried out with 5 repetitive folds by using modified stratified k-fold cross-validation scheme. In the first stage *(i)*, we trained three different models *to detect and classify* COVID-19. At this stage, we aimed to evaluate the performance of the proposed architecture and to show the effect of the proposed hexaxial feature mapping process on the success of the classification. For comparison, we trained the AlexNet architecture using ECG hexaxial mapping images (Experiment 1), the proposed architecture using ECG hexaxial mapping images (Experiment 2), and finally the proposed architecture using 2D ECG spectral images (Experiment 3). In the second stage *(ii)*, we trained our proposed model *to predict and diagnose* COVID-19 (Experiment 4). At this stage, we aimed to evaluate the diagnostic value of ECG by distinguishing ECG disorders caused by COVID-19 from other ECGs without COVID-19 findings and diagnose COVID-19 through ECG data. The classification results of all test phases are given in Table 1.

**Table 1:** Performance evaluation results of trained models

		Performance evaluation metrics								
	Folds	ACC	PRE	REC	SPE	F1-S	AUC	Loss	MSE	TT
**Experiment 1** COVID-19 versus No-Findings via AlexNet	Fold-1	91.00	88.68	94.00	88.00	91.26	97.92	0.283	0.090	103.91
	Fold-2	**95.00**	92.45	98.00	92.00	95.15	96.44	0.680	0.050	101.50
	Fold-3	93.00	89.09	98.00	88.00	93.33	97.16	0.451	0.070	101.56
	Fold-4	94.00	94.00	94.00	94.00	94.00	97.30	0.533	0.060	101.47
	Fold-5	**95.00**	94.12	96.00	94.00	95.05	98.60	0.317	0.050	101.44
	**Average**	**93.60**	**91.67**	**96.00**	**91.20**	**93.76**	**97.48**	**0.453**	**0.064**	**101.98**
**Experiment 2** COVID-19 versus No-Findings via proposed architecture	Fold-1	94.00	90.74	98.00	90.00	94.23	97.36	0.721	0.060	105.10
	Fold-2	96.00	92.59	100.0	92.00	96.15	99.60	0.203	0.040	103.41
	Fold-3	97.00	96.08	98.00	96.00	97.03	99.84	0.151	0.030	102.83
	Fold-4	**98.00**	98.00	98.00	98.00	98.00	99.88	0.086	0.020	102.86
	Fold-5	96.00	94.23	98.00	94.00	96.08	99.08	0.300	0.040	101.87
	**Average**	**96.20**	**94.33**	**98.40**	**94.00**	**96.30**	**99.15**	**0.292**	**0.038**	**103.21**
**Experiment 3** COVID-19 versus No-Findings using only 2D ECG spectral images	Fold-1	**84.83**	82.62	88.22	81.44	85.30	93.56	0.348	0.152	532.43
	Fold-2	81.50	78.46	85.68	77.50	81.91	91.65	0.650	0.177	526.35
	Fold-3	81.85	79.08	84.82	77.77	81.85	91.98	0.592	0.173	525.54
	Fold-4	80.17	80.12	82.26	77.89	81.18	87.16	0.785	0.237	528.07
	Fold-5	77.06	76.80	79.53	74.43	78.14	84.75	0.845	0.362	529.78
	**Average**	**81.08**	**79.42**	**84.10**	**77.81**	**81.68**	**89.82**	**0.644**	**0.220**	**528.43**
**Experiment 4** Negative versus Positive via proposed architecture	Fold-1	92.00	88.89	96.00	88.00	92.31	94.08	0.831	0.080	106.95
	Fold-2	**95.00**	92.45	98.00	92.00	95.15	98.04	0.297	0.050	103.28
	Fold-3	91.00	88.68	94.00	88.00	91.26	95.00	0.520	0.090	103.43
	Fold-4	94.00	90.74	98.00	90.00	94.23	94.06	0.663	0.060	103.71
	Fold-5	93.00	92.16	94.00	92.00	93.07	93.70	0.827	0.070	102.23
	**Average**	**93.00**	**90.58**	**96.00**	**90.00**	**93.20**	**94.98**	**0.628**	**0.070**	**103.92**

*Loss* cross-entropy loss, *TT* training time (s), and all ACC, PRE, REC, SPE, F1-S, and ROC-AUC values are given as %

**Experiment 1:** By training the AlexNet architecture using hexaxial mapping images, an average of 93.60% ACC value was achieved. The best training performance was achieved with 95.00% ACC in the 5th fold and 2nd fold. Test ACC values have a standard deviation of $\mp$2.63%. The deviations of ACC changes in each fold are within acceptable limits. Also, the average PRE, REC, SPE, F1-S, and ROC-AUC values were yielded 91.67%, 96.00%, 91.20%, 93.7%, and 97.48%, respectively. The average test loss was calculated as 0.453 and the average MSE was calculated as 0.064. The obtained REC values were equal or higher than SPE values in all folds. This situation implied that the false-positive rate (FPR) was higher than the false-negative rate (FNR). FPR indicates the rate of being marked to have COVID-19, while the individuals did not have COVID-19. It took an average of 101.98 s to train AlexNet using hexaxial mapping images.

**Experiment 2:** By training the proposed architecture using hexaxial mapping images, an average of 96.20% ACC value was achieved. This average ACC value provided a 2.60% better performance compared to AlexNet. The proposed model exhibited an ACC performance of over 96.00% on all folds, and the best performance was at the 4th fold with an ACC value of 98.00%. ACC values obtained in the test phase had only $\mp$1.48% standard deviation. This situation was an indicator of the robustness of the model. Moreover, the average PRE, REC, SPE, F1-S, and ROC-AUC values were achieved 96.20%, 94.33%, 98.40%, 94.00%, 96.30%, and 99.15%, respectively, and where all values performed better than AlexNet in all cases. The test loss proved the robustness of the model with a small value of 0.292 and a very low MSE of 0.038. By using the proposed architecture, the training time of hexaxial mapping images took only an average of 103.21 sec. An almost ideal classification success has been achieved in Fold-4 with a ROC-AUC value of 99.88%. Similar to the training of AlexNet, FPR values were higher than FNR values. The achieved success in all folds of the proposed method has provided a significant improvement compared to the AlexNet. Furthermore, although the proposed architecture included more layers compared to AlexNet, an average training time difference was only 1.23 sec. Therefore, we used the proposed architecture to train other models.

**Experiment 3:** In this step, we trained our proposed model with 2D ECG spectral images and evaluated the results to observe the success of the proposed hexaxial mapping approach. In this step segmented and pre-processed 2D ECG spectral images were given directly to the deep network as an input. All 18-lead (6 of them augmented) ECG images of each patient were used in the training phase in order to include the information of all ECG channels. There was no evidence that the abnormalities in ECG caused by COVID-19 can be separated on a channel basis. All findings in the studies summarized in the Related works section have been observed on the entire ECG, and as far as we know, no channel-based study has been conducted. Consequently, at each training, validation, and testing phase 7200, 1800, and 1800 2D ECG spectral images were used. As seen in Table 1, by training the proposed architecture with the 2D ECG spectral images, an average of 81.08% ACC was yielded. The highest ACC value was calculated as 84.83% at the 1st fold and ACC values had a standard deviation of $\mp$2.82%. The highest standard deviation occurred at this step. Moreover, the lowest average PRE, REC, SPE, F1-S, and ROC-AUC values were calculated as 79.42%, 84.10%, 77.81%, 81.68%, and 89.82%, respectively in this step. The average test loss was 0.644 and the MSE was 0.220 and was relatively higher than other trained models. Especially, SPE had the lowest value with 77.81%. Due to the increasing training size, the computational cost had increased and the average training time was calculated as 528.43 sec. A significant difference of 15.12% ACC was observed compared to training of hexaxial mapping images. As a result, the hexaxial mapping approach achieved higher performance with less computational cost and training time compared to the training of 2D ECG spectral images.

**Fig. 7:**
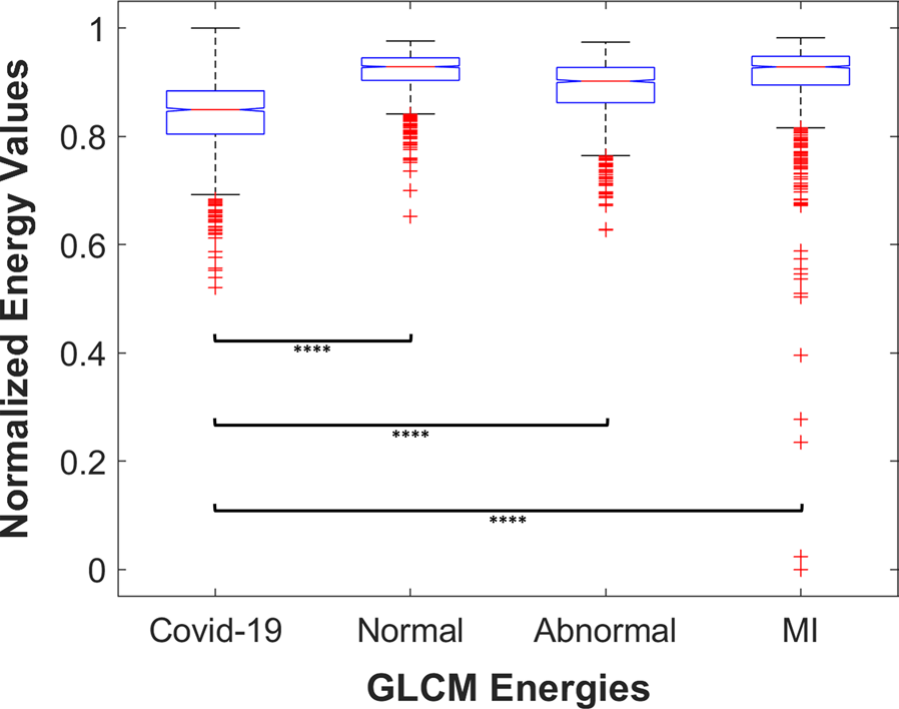
One-way ANOVA results for Negative and Positive comparison within a box plot using COVID-19 GLCM energies and GLCM energies of other ECG groups. Normalized COVID-19 GLCM energies obtained from binary ECG images were verified to statistically significant to each ECG group ($p < 0.0001$ for all cases). Each group has a total of *1494 samples: 18-lead x 83 paper-based ECG reports*

**Experiment 4:** To predict COVID-19 from ECG, two groups were generated Positive versus Negative. While the Positive group consisted of only the ECG data of COVID-19 patients, we included an approximately equal amount of normal ECG (of individuals without any cardiac findings), history of MI patients’ ECG, and abnormal ECG (of patients recovered from COVID-19 or MI) to the Negative group. Firstly, we analyzed the GLCM energy features of both groups statistically by applying a one-way ANOVA test and it verified that the GLCM energy values of COVID-19 ECGs were statistically significantly different from the GLCM energy values of the normal, MI, and abnormal ECGs groups ($p < 0.0001$). The ANOVA results are shown in the Fig. 7. In order to evaluate the success of our proposed hexaxial mapping approach in this classification problem, mapping images that belongs to Positive and Negative groups were trained with the proposed architecture. As seen in Table 1, an average of 93.00% ACC value was achieved with the proposed method and the best ACC value was obtained as 95.00% at the 2nd fold. Test ACC values had a standard deviation of $\mp$1.58%. Moreover, with the proposed approach, the average PRE, REC, SPE, F1-S, and ROC-AUC values were achieved 90.58%, 96.00%, 90.00%, 93.20%, and 94.98%, respectively. Although the average test loss was relatively high (0.628), the MSE value was quite low (0.070). As with other trained models, the FPR value was higher than the FNR value. It took an average of 103.92 s to train the proposed model with the proposed approach.

**Fig. 8:**
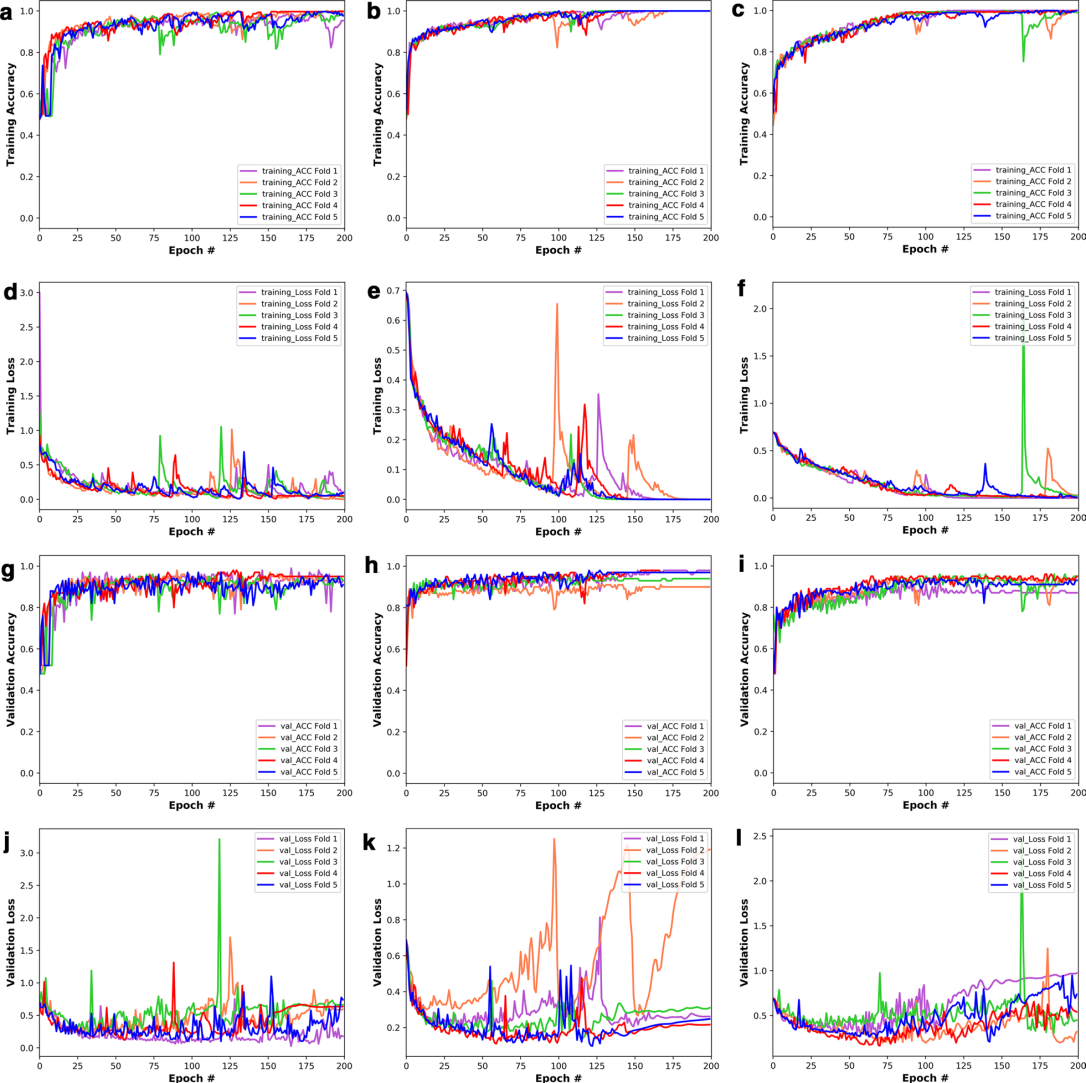
Graphs of training ACC (**a**–**c**), training Loss (**d**–**f**), validation ACC (**g**–**i**), and validation Loss (**j**–**l**) per Epoch during the training and validation phases. The **left column** indicates trained with Alexnet for *COVID-19 versus No-Findings* classification, the **mid column** indicates trained with modified Alexnet for *COVID-19 versus No-Findings* classification, and the **right column** indicates trained with modified Alexnet for *Negative vs Positive* classification

**Fig. 9:**
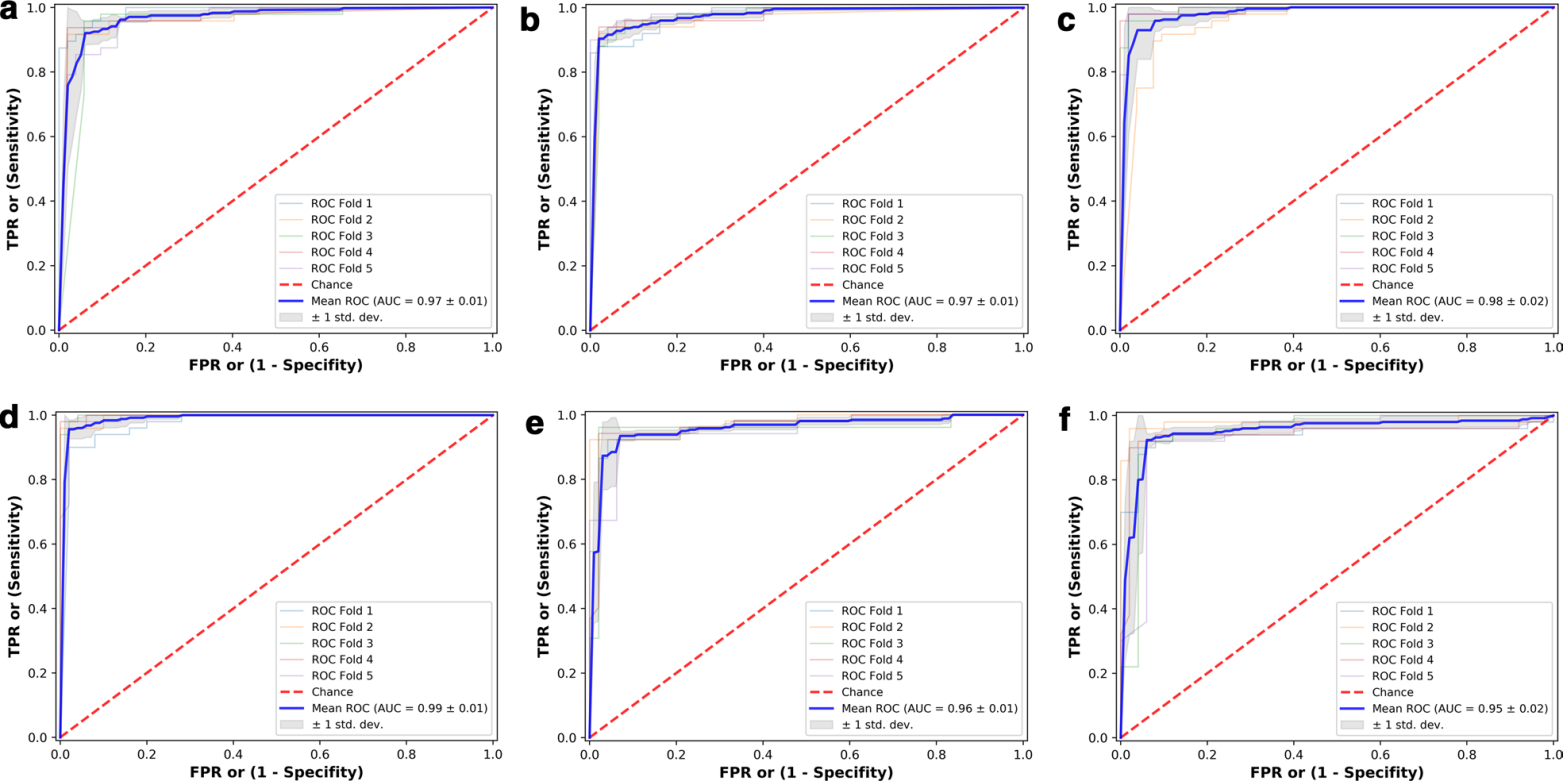
The ROC curves of *COVID-19 vs No-Findings* classification using AlexNet during **a** validation phase and **b** testing phase, using proposed architecture during **c** validation phase and **d** testing phase, and the ROC curves of *Positive versus Negative* classification using proposed architecture during **e** validation phase and **f** testing phase

**Fig. 10:**
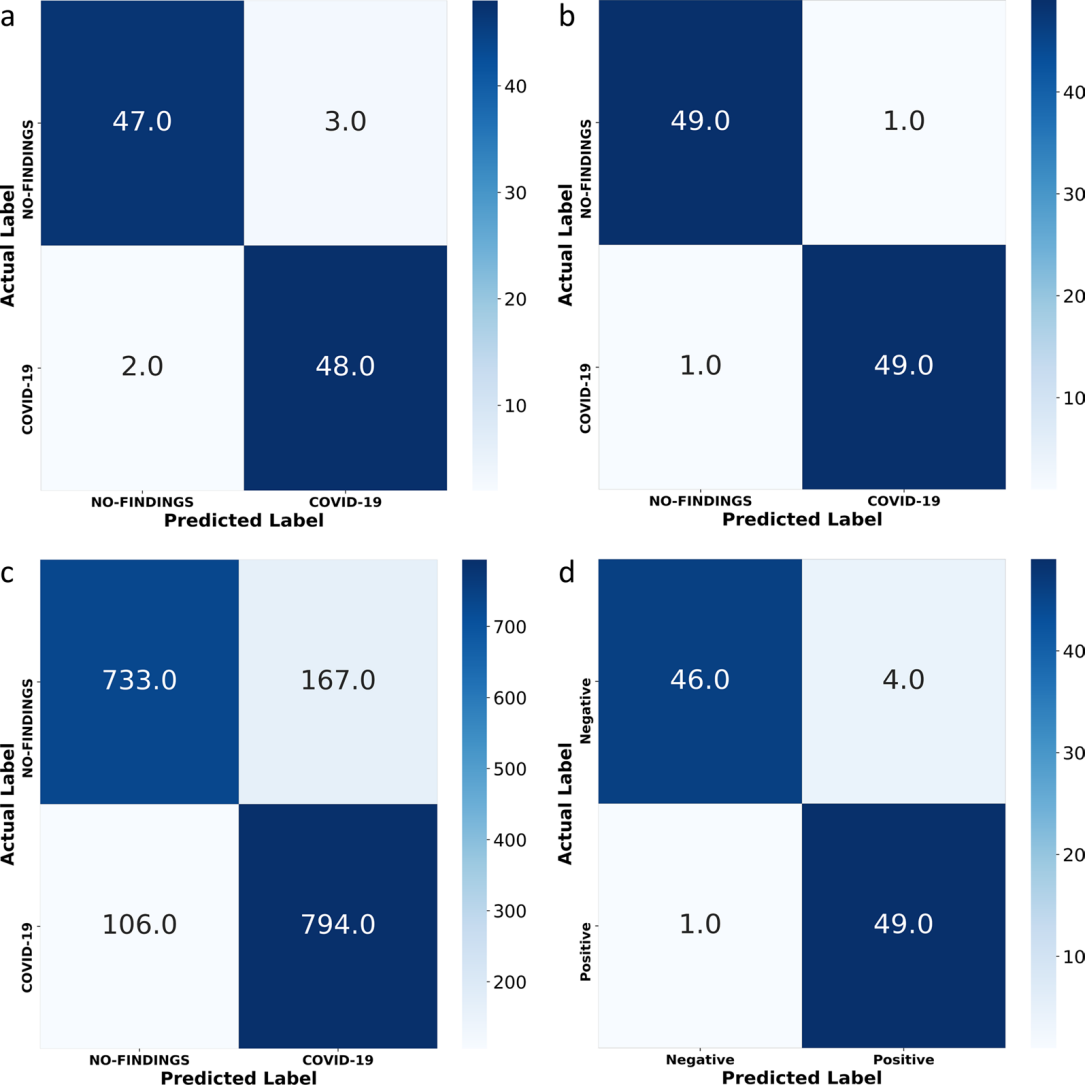
The best confusion matrices obtained during the testing phases: **a** fold-5 in Experiment 1 (*COVID-19 vs. No-Findings*), **b** fold-4 in Experiment 2 (*COVID-19 vs. No-Findings*), **c** fold-1 in Experiment 3 (*COVID-19 vs. No-Findings*), and **d** fold-5 in Experiment 4 (*Positive vs. Negative*)

As mentioned above, the classification of COVID-19 ECG data with the proposed method has yielded outstanding test performance. Further, in Fig. 8 training and validation ACC graphs and training and validation loss graphs are shown for all folds to evaluate both training and validation phases. In all cases for all folds; training ACC and validation ACC values converge to the upper limit. Nevertheless, AlexNet has more volatility and has had difficulty converging compared to the proposed architecture. Moreover, the proposed architecture converged before the 200th epoch. Training loss values converged to the lower limit. Similarly, the proposed architecture converged to the lower limit before the 200th epoch. Due to the dropout method, some temporary loss increases were observed, but they disappeared towards the last epoch. Similarly, the validation loss values converged to the lower limits. However, only in the proposed architecture, although the 2nd fold loss tended to increase, it moved within lower values compared to AlexNet. Also, overfitting or underfitting was not observed in any of the trained models. In the COVID-19 versus No-Findings classification, during the training of AlexNet architecture, average training ACC, training loss, validation ACC, and validation loss were calculated as 98.20%, 0.057, 93.4%, and 0.563, respectively and the proposed model was achieved 100.00%, 0, 96.20%, and 0.269, respectively. Besides, in the Positive versus Negative classification, the proposed model was achieved 99.60%, 0.013, 92.60%, and 0.603, respectively. In order to clearly evaluate the performance of the trained models, the ROC curves for the validation and testing phase of the trained models are given in Fig. 9. As can be seen in the figure, the AUC values for mean ROCs were calculated as the lowest 95% and had a deviation of most $\mp$0.02%. Especially, the ROC curve during the testing phase of the proposed model was almost ideal. Moreover, for this purpose, the best confusion matrices (CM) obtained in the test phase are given in Fig. 10. While there were 5 misclassified labels using AlexNet in Experiment 1, 98 of 100 mapping images were correctly classified by the proposed architecture and only one COVID-19 case was misclassified in Experiment 2. Besides, as seen in CM obtained using only 2D ECG spectral images in Experiment 3, the rate of misclassification was high. Further, even though misclassification performance increased in the CM obtained in Experiment 4, it misclassified only one COVID-19 case.

### Comparison with recent studies

Recently, presented studies to automatically diagnose COVID-19 with deep learning have emphasized well-known architecture ResNet [72]. Accordingly, in addition to the experiments performed, generated hexaxial mapping images were trained with ResNet-50 architecture to compare with our proposed architecture. However, overfitting was observed during the training phase of the models. Therefore, sufficient performance could not be achieved during the testing phase of these models (average 70% ACC). The main reason for this was to train the deeper network with an insufficient number of labeled data. Since sufficient performance was not achieved with our input data in architectures that had more layers, the number of layers of the proposed model was not increased further.

In this study, we want to draw particular attention to the results of Experiment 4 which reveals that ECG may be a diagnostic tool for COVID-19. In fact, in all conducted statistical analyses of GLCM features, a significant difference was observed between ECGs of COVID-19 and the others; MI, abnormal, and no cardiac findings in spite of having low-resolution images and the restricted number of data. Undoubtedly, we would like to point out that we need more ECG data especially ECGs of mild or asymptomatic COVID-19 patient’s to prove our claim. We hope the health science community will share more data on COVID-19.

Additionally, many deep learning-based studies have used radiographic images for the detection of COVID-19 and many of them have achieved outstanding classification performance. The following studies can be shown as an example: Al-Waisy et al. [72] achieved accuracy of 99.99%, Dhiman et al. [79] achieved accuracy of 98.54%, Ozturk et al. [14] achieved accuracy of 98.08%, and Ahuja et al. [74] achieved accuracy of 99.4%. The main reason for the success of the mentioned studies is that the most common symptom of COVID-19 disease is lung involvement [80] and the symptoms can be clearly observed on radiographic lung images [81]. Despite this, some studies using CT and X-ray to diagnose COVID-19 have achieved less accuracy rate than our proposed method. The following studies can be shown as an example: Ismael and Şengür [70] achieved accuracy of 94.7%, Pathak et al. [82] achieved accuracy of 93.02%, Song et al. [69] achieved accuracy of 86%, Amyar et al. [17] achieved accuracy of 94.67%, and Wang et al. [83] achieved accuracy of 82.9%. Moreover, considering the disadvantages of radiological images mentioned in the Background section, the proposed ECG-based COVID-19 diagnosis method may be more useful than the radiological image-based detection methods. In particular, it can be noted that the ECG is more accessible and harmless than CT or X-ray.

Furthermore, many studies are presented to classify cardiac arrhythmias using multi-lead ECG [44, 84]. Arrhythmias may not be observed in all ECG channels and may be dominant only in some ECG channels. Especially in multi-lead ECG and AI-based classification studies, all channel information should be protected. Otherwise, an abnormal ECG may be misclassified if the prediction is performed through the ECG channel where no abnormality is observed. Since the proposed hexaxial mapping method includes all 12-lead channel information, no channel in which arrhythmias can be observed has been ignored. Moreover, the proposed hexaxial mapping method supports the representation of not only paper-based ECG images but also 2D spectral images of digital ECG signals. Therefore, it can be used in the representation and classification of cardiac arrhythmias from digital ECG signals and can be an alternative to current automated arrhythmia detection approaches.

### Major contribution of the study

The COVID-19 pandemic has caused many medical challenges. A fast and easily accessible method is required for the early and accurate diagnosis of the disease. Detection of COVID-19 with ECG data using a deep learning approach shows promise as a new diagnostic method. In this respect, this paper makes several contributions to the literature. These innovative contributions may be emphasized as follows:

- A novel, highly sensitive, and harmless method has been proposed as an alternative to the existing diagnostic methods to aid in the diagnosis of COVID-19.
- A new and effective approach has been proposed in order to classify paper-based ECG data, where all ECG-leads can be represented as a single colorful 2D image.
- Differences in the ECG data of patients with COVID-19 and individuals without any cardiac findings and patients with various arrhythmias were demonstrated.
- The experimental classification results can be evidence for the presence of cardiovascular changes caused by COVID-19.
- The advantages of the proposed hexaxial feature mapping process on classification performance were demonstrated.
- A new and simple deep network architecture has been proposed for 2D image classification and the deep network hyperparameters were optimized to yield the best classification performance.

### Limitations and future scope

Nonetheless, some limitations should be noted. In particular, the hexaxial feature mapping process is very sensitive to the resolution of paper-based ECG images. Resolution variations in ECG images may cause differentiation in the features obtained through GLCM and may affect the color intensity of hexaxial maps. Further, while performing the segmentation of ECG-lead images, the size of the selected rectangular frame must be kept constant. It should be noted that the segmentation process can be standardized by using a smart-phone application that guides the user for taking the right ECG image from the paper-based ECG report. Additionally, although the proposed method is designed as a patient-independent approach and its robustness has been tested with various experimental scenarios, it needs to be evaluated with different datasets. The main limitation here is the lack of access to the COVID-19 patients’ ECG data and the lack of a sufficient amount of data. Moreover, the dataset in which the proposed method is tested does not contain any information about the severity of the condition of COVID-19 patients. This prevents an evaluation of the occurrence of COVID-19-induced cardiovascular changes.

Another limitation is that there may be variability in the number of leads and derivation when collecting ECG data. Although the proposed method requires 12 basic leads, ECG data collected from various derivations can be adapted to the hexaxial mapping process. It should also be noted that this work aims to discuss the ability to automatically distinguish COVID-19 ECG data from other types of ECG data. Although recent studies [30, 32–34, 53] have reported various cardiovascular changes in most of the patients, they also reported infected patients without any cardiovascular changes. Therefore, the sensitivity of the proposed method is related to the observability of cardiovascular changes. Furthermore, there are concerns that COVID-19 may not be the main source of cardiovascular changes in ECG data [25]. Thus, two main issues could be addressed in future research; further research should be attempted to specify COVID-19-induced cardiovascular changes, and the current method should be tested on a more robust dataset.

## Conclusion

In this study, a novel and effective approach is proposed to automatically detect COVID-19 using paper-based ECG report images. This study aims to distinguish the ECGs of COVID-19 patients from various types of ECGs. Accordingly, a novel method based on representing 12-lead paper-based ECG images as 2D colorful images has been proposed and the generated colored images are then fed into a new CNN architecture to detect COVID-19. While recent state-of-the-art studies have revealed that COVID-19 can lead to cardiovascular complications directly or indirectly, ECG data is used for the first time to automatically diagnose COVID-19, to the best of our knowledge.

Various experiments are conducted to evaluate the robustness of the proposed approach and compare its performance. The results demonstrated that the proposed method achieved promising performance in the diagnosis of COVID-19 using ECG data. Furthermore, the proposed deep network significantly improved classification accuracy compared to well-known architectures and the proposed hexaxial mapping procedure not only decreased computational cost, but it also significantly increased classification performance. Furthermore, the capability of the proposed approach to differentiate COVID-19 ECGs can be the proof of the presence of COVID-19-induced cardiovascular changes.

In the light of all findings, we can say that; the proposed approach can potentially be used as a faster, more harmless, more accessible, cost-effective, and more sensitive automatically diagnostic method to detect COVID-19 than the current methods. In future works, the presented ECG-based COVID-19 diagnosis method can be simply adapted to real-time cloud-based systems and can be easily performed on mobile device-based decision-making applications. Thus, it may help healthcare professionals by providing a fast and effective solution to diagnose COVID-19, it may reduce both the contamination and the hospital burden by preventing unnecessary hospital visits.

## Data Availability

Python and MATLAB source codes, and segmented and preprocessed dataset are available from: https://github.com/mkfzdmr/COVID-19-ECG-Classification. The dataset used in this work is publicly available at: http://dx.doi.org/10.17632/gwbz3fsgp8.1

## Abbreviations

ACC: accuracy
AF: atrial fibrillation
AI: artificial intelligence
ANOVA: analysis of vatriance
CM: confusion matrices
CNN: convolutional neural network
Conv: convolution
COVID-19: coronavirus disease 2019
CT: computed tomography
CWT: continuous wavelet transform
ECG: electrocardiogram
F1-S: F1-score
FNR: false-negative rate
FPR: false-positive rate
GLCM: gray-level co-occurrence matrix
ILSVRC2012: Large Scale Visual Recognition Challenge in 2012
IoT: Internet of Things
MI: myocardial infarction
MSE: mean squared error
PRE: precision
RBBB: right bundle branch block
REC: recall
ReLU: rectified linear units
ROC-AUC: area under the receiver operating characteristic curve
rRT-PCR: real-time reverse transcriptase-polymerase chain reaction
RVPO: right ventricular pressure overload
SARS-CoV-2: severe acute respiratory syndrome coronavirus 2
SPE: specificity
STFT: short-time Fourier transform
SVM: support vector machines
WHO: World Health Organization
